# The Impact of Visual Aids and Enhanced Training on the Delivery of Positive Health, Dignity, and Prevention Messages to Adult Patients Living with HIV in Rural North Central Mozambique

**DOI:** 10.1371/journal.pone.0130676

**Published:** 2015-07-06

**Authors:** Carolyn M. Audet, Sarah A. Gutin, Meridith Blevins, Elvino Chiau, Fernanda Alvim, Eurico Jose, Lara M. E. Vaz, Bryan E Shepherd, Carol Dawson Rose

**Affiliations:** 1 Vanderbilt University School of Medicine, Nashville, Tennessee, United States of America; 2 Departments of Health Policy, Vanderbilt University School of Medicine, Nashville, Tennessee, United States of America; 3 Biostatistics, Vanderbilt University School of Medicine, Nashville, Tennessee, United States of America; 4 Community Health Systems, School of Nursing, University of California San Francisco, San Francisco, California, United States of America; 5 Friends in Global Health, Maputo and Quelimane, Mozambique; 6 Vanderbilt Institute for Global Health, Vanderbilt University, Nashville, Tennessee, United States of America; State University of Maringá/Universidade Estadual de Maringá, BRAZIL

## Abstract

**Introduction:**

Positive health, dignity, and prevention (PHDP) interventions target people living with HIV and AIDS (PLHIV) to promote well-being and prevent onward transmission. Concern that increased life expectancy and improved well-being would lead to increased risky sexual behaviour and subsequent HIV transmission motivated researchers to test novel strategies to support treatment adherence, encourage safer sex, STI treatment and partner testing, prevention of mother to child transmission, and support uptake of family planning.

**Methods:**

We assessed the number and type of PHDP messages delivered to PLHIV before and after the implementation of an educational intervention for health providers combined with the distribution of visual job aids and monthly technical assistance.

**Results:**

From April 21, 2013 to March 20, 2014, we documented 54,731 clinical encounters at three rural health centres in Zambézia province, Mozambique from 9,248 unique patients. The percentage of patients who received all seven PHDP messages during their last three visits was 1.9% pre-intervention vs. 13.6% post- intervention (p=<0.001). Younger patients (25 years vs. 35) and those with a recent HIV diagnosis (two weeks vs. two years) had higher odds of receiving any PHDP message (Odds Ratio [OR]: 1.22 and 2.79, respectively). Patients >59 days late collecting medications were not more likely to receive adherence messages than adherent patients (p=0.17).

**Discussion:**

Targeting HIV prevention efforts to PLHIV is an effective HIV prevention approach to eliminate HIV transmission. Despite intensive training and support, PHDP message delivery remained unacceptably low in rural Mozambique. Patients at high risk for treatment abandonment were not more likely to be counselled about adherence and support measures, something that needs to be addressed.

**Conclusions:**

We need to develop novel strategies to motivate health care providers to deliver these messages more consistently to all patients and develop a system that assists counsellors and clinicians to quickly and effectively determine which messages should be delivered.

## Introduction

Mozambique has one of the world’s highest burdens of human immunodeficiency virus (HIV) and acquired immunodeficiency syndrome (AIDS), with an estimated 11.1% of the adult population living with HIV [1]. The Mozambican Ministry of Health (MISAU) has partnered with the Centers for Disease Control and Prevention (CDC) to provide nationwide free access to HIV care and treatment. Given the generalized HIV epidemic, the current national strategy is the antiretroviral therapy (ART) acceleration plan, which aims to have 85% of individuals who are HIV infected, started on antiretroviral medication as directed through the World Health Organization (WHO) HIV treatment guidelines [2]. Initiation, retention, and adherence to ART reduces the risk of HIV transmission to sexual partners and infants [3, 4] and mortality [5]. As a result, life expectancy for those initiating treatment with baseline CD4 counts >350 cells/mm^3^ is approaching that of the general population [6]. Concern that increased life expectancy and improved well-being would lead to increased risky sexual behaviour and subsequent HIV transmission [7–11] motivated researchers to test novel clinical strategies to support treatment adherence, encourage safer sex, STI treatment and partner testing, and support uptake of family planning services [12, 13].

Positive health, dignity, and prevention (PHDP), or as they are known in Mozambique, Positive prevention (PP) interventions, specifically target people living with HIV and AIDS (PLHIV) to promote well-being and prevent onward transmission, including sexual transmission and mother-to-child transmission [14–20]. Strategies to improve PHDP behaviours have included: clinical based couples counselling and testing,[21–25] counselling and increased availability of family planning,[26] individualized clinician-delivered prevention interventions,[27, 28] and group counselling and education for HIV-infected adults;[29] internet or computer-based education sessions; [30] and peer-delivered HIV prevention interventions [31]. Most studies have shown that communication of prevention messages is effective at increasing condom use and uptake of family planning services when done by a health care worker (HCW) in a clinic setting [32–35]. Mozambique is one of the few countries that has prioritized PHDP as part of their national strategy to address HIV prevention in all health facilities. The approach in Mozambique is to train and enlist all health facility staff to ensure patients receive messages throughout the continuum of care. However, two primary challenges limit scale up of PHDP: (1) low-resourced health infrastructure and staffing to meet the population demands; and (2) extremely low health literacy levels among patients, estimated at 52% nationwide [36].

Training HCWs to incorporate PHDP message delivery and counselling into their clinical services presents a solid, evidence-based approach to the prevention of HIV transmission by those already infected [35]. Integrating PHDP into clinical service delivery in rural Mozambique has remained a challenge, however, despite the introduction of an evidence-based curriculum by the University of California, San Francisco (UCSF)[37, 38]. While PHDP training materials and sessions were acceptable to both patients and providers,[37] delivery of PHDP messages remained poorly documented in the medical record. The purpose of this study was to assess the number and type of PHDP messages delivered to PLHIV before and after the implementation of an educational intervention for clinicians providing HIV care and treatment combined with the distribution of job aids (including posters and flipcharts with images depicting PHDP messages) to each clinical and counselling room.

## Materials and Methods

### Study Setting

Morrumbala, Pebane and Maganja da Costa are rural districts with estimated populations of 215,000 (Morrumbala and Pebane) and 305,000 (Maganja da Costa) respectively. Educational attainment in the region is typically less than primary school and few people have any monthly income [39]. The three district hospitals included in the study provide HIV testing, care and treatment services (including ART), prevention of mother-to-child transmission (PMTCT), family planning, and early infant diagnosis and treatment, free of charge. These sites were chosen for their linguistic variability (they represent three distinct linguistic groups) and because they provide all referral services described on the PHDP package of services

### Study Population

All HIV-positive male and female patients aged ≥ 18 years with at least one contact (clinical appointment, counselling session, or pharmacy pick-up) at one of the three study sites from April 2013 to March 2014 were included in the study. Patients < 18 years of age enrolled in care were excluded.

### Positive Prevention Intervention Protocol

**Fig 1:**
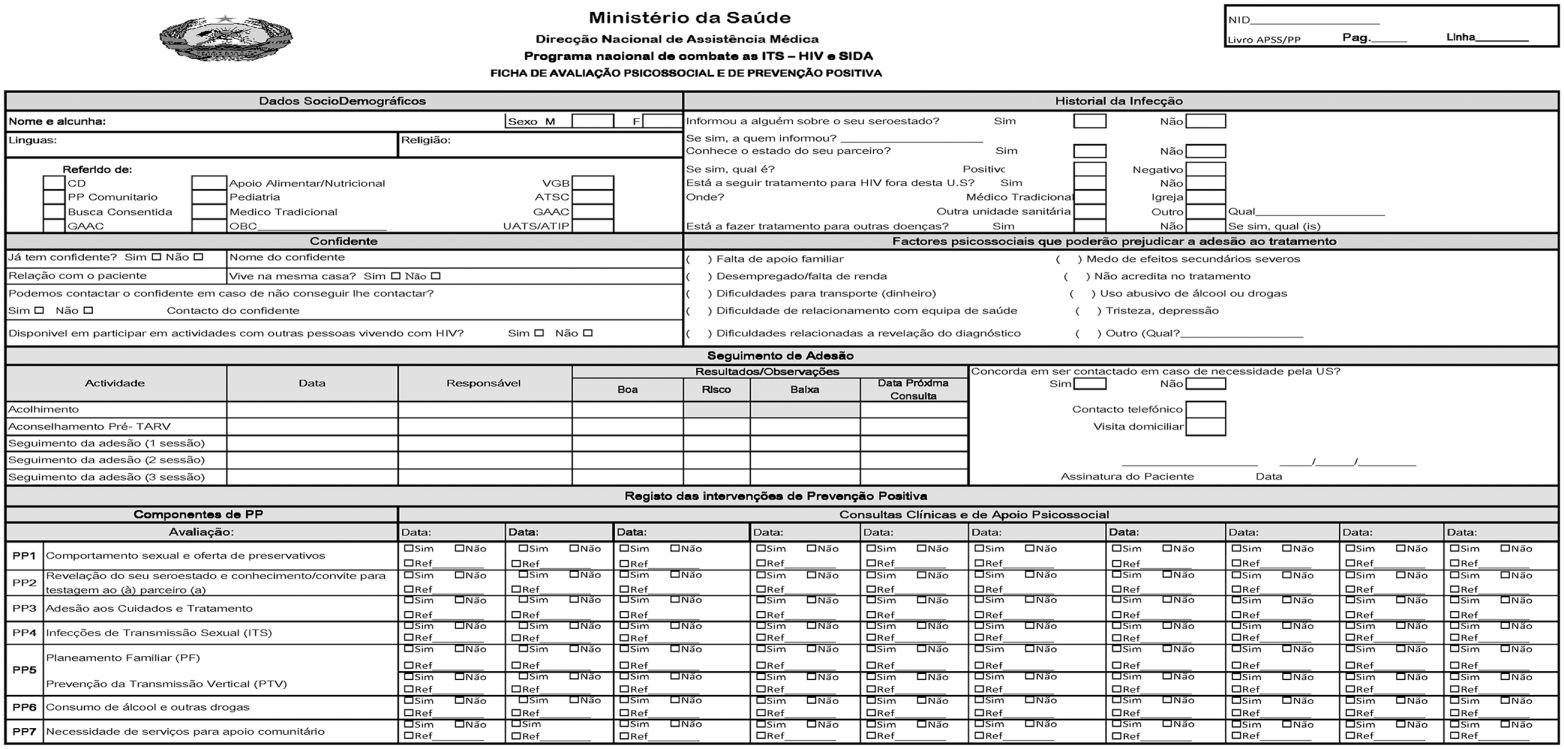
Psychosocial Evaluation and Positive Prevention form.

Every patient receiving HIV care and treatment in Mozambique should be provided PHDP messages during clinical, counselling, and pharmacy visits [40]. These messages (part of the Positive Prevention Toolkit) were developed by researchers at University of California, San Francisco [41], from pilot studies in Maputo, Mozambique [37, 38, 42] and implemented as a national strategy by the Ministry of Health in April 2013. Concurrently, a new form, the *Ficha de Avaliação Psicossocial e de Prevenção Positiva* (Psychosocial Assessment and Positive Prevention Record) was introduced into patient records to allow providers to document delivery of PHDP messages during each clinical encounter (Fig 1). In November 2013, a four day short-course PHDP training session for all HIV care and treatment clinicians and counsellors was provided at the three study sites. The training focused on the importance of PHDP, how and when to deliver messages, how to address patient barriers to service uptake, and how to use new visual aids (a poster and flip-chart containing images of PHDP services and behaviours). These providers were also given a PHDP poster and flip-chart for their offices which provide visual images of PHDP messages to facilitate discussion with patients. Monthly technical assistance follow-up visits at the HCW’s clinical care site within four weeks of the training were provided to improve the implementation of the PHDP intervention in their day-to-day work and as a way to provide reinforcement for the skills taught in the enhanced PHDP training package. Providers were trained to ensure that each patient received all seven messages (including the most relevant PHDP 5 message) every three months (Table 1).

**Table 1:** Positive Health, Dignity, and Prevention (PHDP) messages.

PHDP1	Sexual behavior and use of condoms
PHDP2	Reveal serostatus to partner/invite partner to test
PHDP3	Adherence to care and medication
PHDP4	Screening for sexually transmitted infections
PHDP5a	Family planning service availability
PHDP5b	PMTCT
PHDP6	Use of drugs and alcohol
PHDP7	Community support group referral

### Data Management and Outcomes

Data was documented during each patient visit by the provider using the *Ficha de Avaliação Psicossocial e de Prevenção Positiva* (a Ministry of Health approved form) and subsequently entered into an OpenMRS Electronic Medical Records (EMR). Major categories of data included: demographics (age, sex, marital status, service entry into program), routine clinical information (pregnancy status, partner serostatus, use of ART, and last two pharmacy pick-ups), and receipt of the seven PHDP messages at each clinical visit. Of primary interest was the number of messages received. Additionally, type of message receipt was considered. The number and type of messages received occurs at the visit level and the database includes anywhere from 1 to 21 visits per patient. Messages are detailed in Table 1.

### Ethics considerations

This study was approved by the Vanderbilt University Institutional Review Board, the University of California, San Francisco Committee on Human Research, the Comité Nacional de Bioética a Saúde in Mozambique, and the Centers for Disease Control and Prevention in Maputo, Mozambique and Atlanta, USA. The data utilized constitute fully de-identified data; the research file has an encrypted identifier and does not include patient names, addresses, or ID numbers. As the data was de-identified when it was collected, patient consent was not required.

### Statistical methods

Patient characteristics were compared among those with visits before and after intervention using descriptive statistics. The proportion of patients who received all seven PHDP messages during their last three visits (excluding pharmacy-only visits) was compared using chi-square test. This analysis was repeated for patients who received four of seven messages during the same time period. The frequency and proportion of visits with PHDP message receipt were compared before and after intervention using cluster-adjusted Wald statistics to account for correlation between visits from the same patient.

Multivariable negative binomial regression with robust variance estimation was used to determine whether the incidence of message receipt was higher post-training. Multivariable logistic regression with robust variance estimation modelled receipt of any PHDP message as a binary response variable. Both models included an indicator for pre-/post-training and adjusted for health facility, time since HIV diagnosis, patient sex, and age. We hypothesized that message receipt across patient sex would be different following training and that sites would have differential PHDP message delivery following training; thus, a three-way interaction effect was included in both models for pre-/post-training, health facility, and patient sex. The robust covariance estimates account for correlation of the count data across multiple patient visits. To relax linearity assumptions in the logistic regression, we modelled age and time since HIV diagnosis using restricted cubic splines with 4 knots [43]. The database had a record for date of last scheduled ART pickup and last actual ART pickup. We subsetted to each patient's last visit occurring after the last pharmacy pick-up and consider only PHDP3 as the outcome of interest, such that we modelled the probability of adherence message receipt using logistic regression. The model included an indicator for late pharmacy pick-up (>59 days late) and adjusts for pre-/post-intervention receipt, health facility, time since HIV diagnosis, patient sex, and age. Additionally, we tested for an interaction between pre-/post-training and site to determine if administration of the adherence message differed across site. To relax linearity assumptions, we modelled age and time since HIV diagnosis using restricted cubic splines with 4 knots [43]. Marital status was missing for nearly 40% of patients and was excluded from regression models. All regression models used complete cases with no imputation; missing data was minimal. All statistical analyses were conducted using R-software 3.0.2 (www.r-project.org/).

## Results

**Fig 2:**
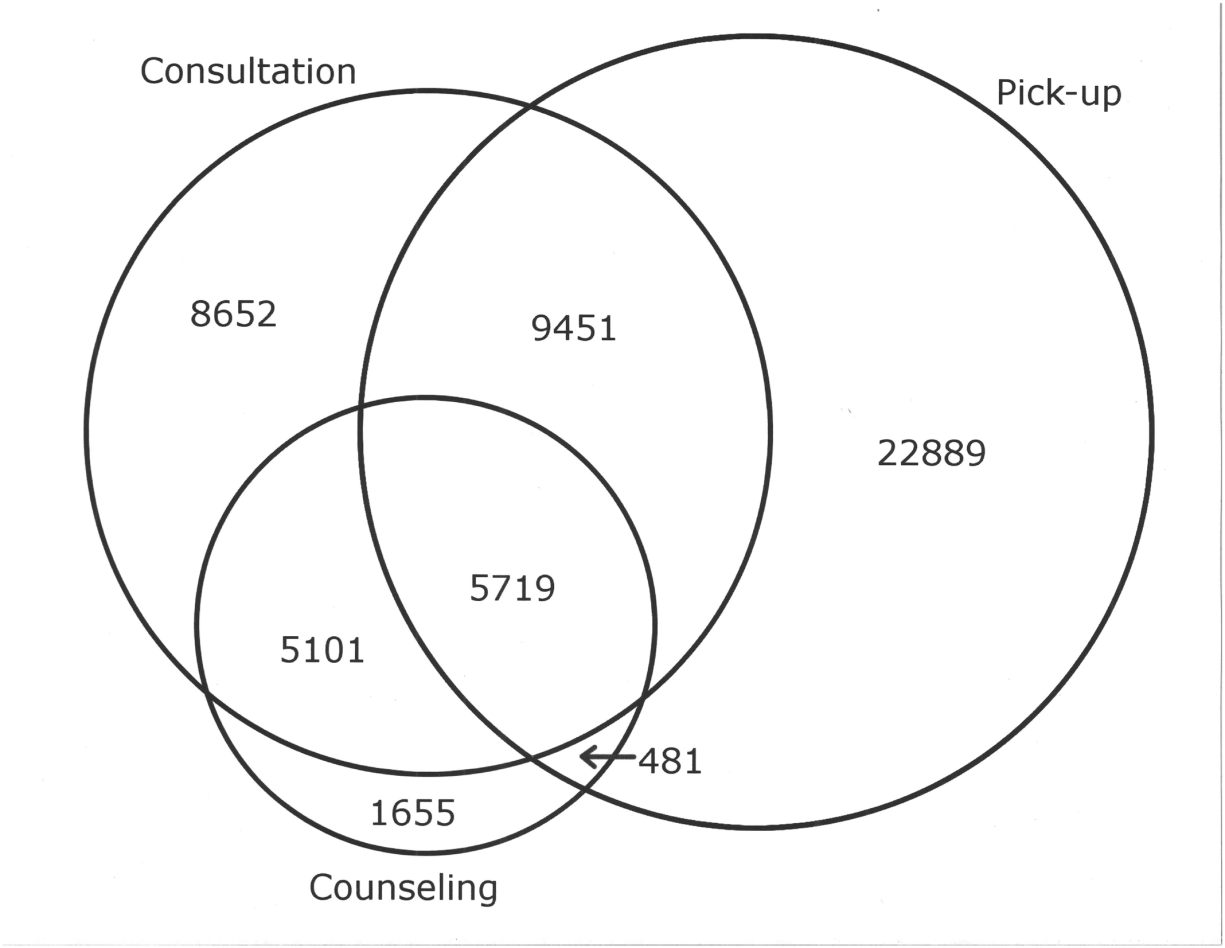
Types of Visits during Study Period.

From April 21, 2013 to March 20, 2014, we documented 54,731 clinical encounters at Maganja da Costa, Pebane, and Morrumbala from 9,248 unique patients. The number of visits per patient during follow-up ranged from 1 to 21. Table 2 summarizes characteristics of eligible participants. Participants were primarily female (70%), had a median age of 32 years (interquartile range [IQR]: 26–39), were taking ART (77%), and were living with a partner (38%). Patients received up to three types of services at each visit: (1) medical consultation; (2) medication pick-up; and (3) psychosocial counselling. Fig 2 shows a diagram: types of visits during the study.

**Table 2:** Summary of Patient Characteristics.

	Maganja da Costa	Pebane	Morrumbala	Combined
	(n = 3405)	(n = 3157)	(n = 2686)	(n = 9248)
Female, n(%)	2501 (73%)	2132 (68%)	1814 (68%)	6447 (70%)
Current age^b^, median (IQR)	31 (26–38)	32 (26–41)	32 (26–39)	32 (26–39)
Age at enrollment, median (IQR)	29 (23–35)	30 (24–39)	29 (24–36)	29 (23–37)
Marital status, n(%)				
Missing	1023 (30%)	1465 (46%)	1110 (41%)	3598 (39%)
Divorced	3 (< 1%)	1 (< 1%)	0 (0%)	4 (< 1%)
Living with partner	1405 (59%)	476 (28%)	291 (18%)	2172 (38%)
Married	341 (14%)	590 (35%)	735 (47%)	1666 (29%)
Never married	449 (19%)	535 (32%)	423 (27%)	1407 (25%)
Separated	1 (< 1%)	2 (< 1%)	2 (< 1%)	5 (< 1%)
Widowed	183 (8%)	88 (5%)	125 (8%)	396 (7%)
Currently pregnant^b^, n(%)	397 (12%)	205 (6%)	244 (9%)	846 (9%)
Currently on ART^b^, n(%)	2552 (75%)	2389 (76%)	2178 (81%)	7119 (77%)
Time since HIV diagnosis (days), median (IQR)	399 (0–1220)	355 (0–1010)	528 (14–1430)	418 (0–1210)
Missing, n(%)	99 (3%)	175 (6%)	120 (4%)	394 (4%)
GAAC (community adherence groups)	310 (9%)	22 (1%)	456 (17%)	788 (9%)
Current visit type^b^ ^,^ ^c^, n(%)				
Consultation	2796 (82%)	1653 (52%)	1881 (70%)	6330 (68%)
Med pick-up	1736 (51%)	1659 (53%)	1497 (56%)	4892 (53%)
Counseling	542 (16%)	1385 (44%)	1505 (56%)	3432 (37%)

Interquartile range (IQR) comprises the 25^th^ and 75^th^ percentiles.

^b^ Current variables reflect data from the first visit during study period.

^c^ Percentages may sum over 100%.

PEPFAR, the CDC and the Mozambican Ministry of Health recommend that each patient receive all seven PHDP messages within three visits to the health facility. The percentage of patients who received all seven PHDP messages during their last three visits pre-intervention was 1.9% vs. 13.6% post- intervention (excluding pharmacy-only visits) (p-value <0.001). We also computed the percentage of patients who received four of seven messages over the same time period (with the assumption that clinicians may already know some messages were not relevant to a particular person based on clinical or social history of the patient). We found that only 14.8% received four messages pre-intervention vs. 38.5% post-intervention (p-value <0.001).

Frequency of PHDP message delivery increased from pre- to post-intervention (Table 3: Positive Prevention Message Receipt). Post-intervention, the most commonly delivered messages were: (1) the importance of adherence to ART (14%); (2) the importance of using condoms during sex (14%); and (3) the importance of revealing serostatus to partner/inviting one’s partner to test (13%). Messages about use of drugs and alcohol, community support groups, and family planning/PMTCT showed the greatest improvements, given extremely low delivery levels pre-intervention.

**Table 3:** Receipt of Positive Prevention Messages during 54731 Patient Visits by Intervention Period.

	Pre	Post	Combined	P-value^a^
	(n = 34041)	(n = 20690)	(n = 54731)	
Sexual behavior and use of condoms	2475 (7%)	2899 (14%)	5374 (10%)	< 0.001
Reveal serostatus to partner/invite partner to test	2043 (6%)	2767 (13%)	4810 (9%)	< 0.001
Adherence to care and medication	2482 (7%)	2887 (14%)	5369 (10%)	< 0.001
Sexually transmitted infections	1296 (4%)	2387 (12%)	3683 (7%)	< 0.001
Family planning	1051 (3%)	1924 (9%)	2975 (5%)	< 0.001
PMTCT	736 (2%)	1718 (8%)	2454 (4%)	< 0.001
Use of drugs and alcohol	773 (2%)	2235 (11%)	3008 (5%)	< 0.001
Community support group referral	503 (1%)	1695 (8%)	2198 (4%)	< 0.001

^a^ Tests of association based on cluster-adjusted Wald statistics that account for correlation between visits from the same patient. There are 9248 patients and 54731 visits included in this table. During pre-intervention, 7702 patients were seen, and during post intervention 6734 patients were seen; among these, 5188 had visits both pre- and post-intervention. The most visits for any one patient pre-intervention was 15 and post-intervention was 13 visits.

Table 4 shows factors associated with the number of messages received. Younger patients and those more recently diagnosed with HIV were more likely to receive messages. Patient visits including a clinical consultation or counselling contact had rates of message delivery almost twice those of other patient visits. Message delivery was much higher after intervention than before, the change differed by sex and site (p<0.001). The rate of message receipt for a female patient in Morrumbala was almost four times greater post-intervention than pre-intervention training. In contrast, the rate of message receipt for a female in Pebane post-intervention was only 1.22 times greater than the rate at pre-intervention.

**Table 4:** Adjusted incident rate ratios for number of messages received in a visit.

	Incident Rate Ratio (95% CI)
Age (per 1 year increase)	0.98 (0.98, 0.99)
Time since HIV diagnosis (per 1 sqrt-day increase)	0.98 (0.97, 0.98)
Consultation	2.01 (1.86, 2.17)
Pharmacy pick-up	0.73 (0.69, 0.77)
Counseling	2.91 (2.72, 3.11)
Contrasts of Post vs. Pre (ref)	
Female at Maganja da Costa	1.84 (1.68, 2.02)
Male at Maganja da Costa	2.59 (2.05, 3.28)
Female at Morrumbala	3.73 (3.16, 4.42)
Male at Morrumbala	3.66 (2.44, 5.47)
Female at Pebane	1.22 (1.01, 1.46)
Male at Pebane	2.48 (1.59, 3.86)

Results were similar when we looked at factors associated with any receipt of messages. Younger patients (25 years vs. 35) and those with a recent HIV diagnosis (two weeks vs. two years) had higher odds of receiving any PHDP message (Odds Ratio [OR]: 1.22 and 2.79, respectively) (Table 5: Adjusted Odds Ratios for ANY PHDP Message Receipt). Those who received counselling had 5.1 times greater odds of receiving a message (vs. those who did not receive counselling that visit), while those who received a clinical consultation had 2.54 times the odds of receiving a message (vs. someone who did not receive a clinical consultation). People who collected medications from the pharmacy had 25% lower odds of receiving a message (vs. those who did not pick up medication). This trend existed for all messages. Delivery of any PHDP message increased for men and women at all three clinics post-intervention. While improvements were noted in each clinic for every type of PHDP message, Pebane consistently had lower odds of increased message delivery than Morrumbala and Maganja da Costa.

**Table 5:** Adjusted Odds Ratios for ANY PP Message Receipt.

	Odds Ratio (95% CI)
Age	
25 years	1.22 (1.15, 1.30)
35 years (ref)	1
45 years	0.91 (0.86, 0.97)
Time since HIV diagnosis	
Recent (two weeks)	2.79 (2.51, 3.11)
One year	1.31 (1.27, 1.35)
Two years (ref)	1
Five years	1.48 (1.35, 1.63)
Consultation	2.54 (2.34, 2.77)
Pharmacy pick-up	0.75 (0.70, 0.80)
Counseling	5.10 (4.71, 5.52)
Contrasts of Post vs. Pre (ref)	
Female at Maganja da Costa	2.01 (1.82, 2.23)
Male at Maganja da Costa	2.61 (2.17, 3.14)
Female at Morrumbala	3.38 (3.02, 3.78)
Male at Morrumbala	3.28 (2.73, 3.94)
Female at Pebane	1.50 (1.29, 1.75)
Male at Pebane	2.49 (1.86, 3.32)

Age and time since diagnosis are modeled using restricted cubic splines with 4 knots.

Tests of association: age (p < 0.001), time since HIV diagnosis(p < 0.001), sex, (p < 0.001), clinic (p < 0.001), intervention (p < 0.001), clinic consultation (p < 0.001), pharmacy pick-up (p < 0.001), counseling visit (p < 0.001), three-way interaction of sex-clinic-intervention (p = 0.017).

We modelled the probability of adherence message delivery to determine if a patient being more than 59 days late for pharmacy visit had higher odds of receiving an adherence message receipt from clinicians. While there were statistically significant increases in adherence message delivery pre- and post-intervention at all sites (data not shown), there was little evidence that those who were non-adherent to their medication pick-up had higher odds of receiving a message about the importance of adherence (OR: 1.23; 95% CI: 0.91–1.65; p = 0.18). Considering adherence message receipt among all adult visits, messages about adherence were most commonly delivered by counsellors (OR: 3.82; 95%CI: 3.51–4.16) and clinicians (OR: 2.33; 95% CI: 2.12–2.57), with pharmacists least likely (OR: 0.73; 95% CI:0.67–0.78) to deliver messages.

## Discussion

While HIV risk reduction interventions for PLHIV have been shown to be effective in the United States [44–47], few programs have focused on developing HIV prevention interventions within clinical settings for PLHIV in African contexts, despite the need for these. One successful program in South Africa has found that PHDP interventions can be delivered with fidelity with counsellors providing the majority of intervention steps during visits [33, 34]. The results of another prevention intervention package for healthcare and treatment settings in Kenya, Namibia, and Tanzania are yet to be released [48, 49]. In Mozambique, in addition to a National ART acceleration plan that seeks to reach 85% of infected Mozambicans with antiretroviral medication, the Ministry of Health is using Positive Prevention/ PHDP, a health education and behavioural approach, towards facilitating better HIV outcomes and less HIV transmissions [40]. In this study in rural Mozambique, we assessed whether a PHDP training approach focused on using updated training materials, support guides and job aides along with monthly HCW technical assistance follow-up visits would improve the delivery of PHDP messages during clinical encounters, as documented in the clinical record. Overall, as a result of the PHDP training and follow-up approach, improvements were seen in both the percentage of patients who received all seven PHDP messages during their last three visits as well as the percentage of patients who received each individual PHDP message, but the proportion of patients receiving adequate PHDP messages remains sub-optimal. Despite intensive training and support, PHDP message delivery remained unacceptably low in rural Mozambique. We need to develop novel strategies to motivate health care providers to deliver these messages more consistently to all patients.

In this study younger patients and those with a recent HIV diagnosis had higher odds of receiving PHDP messages. While we did not investigate provider rational for message delivery focused on these two populations the literature suggests: (1) younger HIV-positive patients engage in higher risk behaviour than older adults; (2) clinicians are more comfortable addressing prevention with younger patients and those new to HIV care; and (3) patients newly-engaging in care have the least information and thus are in need of a greater amount of counselling [50–54]. Several studies correlate younger age with HIV risk behaviour in HIV-positive clinic samples [50–52]. Flickinger found that younger patients and those who had a shorter duration of the patient provider relationship were more likely to report greater sexual risk behaviour when compared with older, more established patients [54]. Duration of the clinical relationship may indicate a more recent diagnosis [53] and HIV-positive patients recently engaged in care report higher risk behaviours [11]. Other studies have indicated that it is important to capitalize on those newly infected patients [55] because diagnosis presents an opportunity to address prevention of transmission earlier in their illness. Offering continued messages to patients who have been living with HIV and engaged with services however is also necessary as these patients will continue to have distinct and changing needs over time [7, 56]. In addition, PHDP is not only about decreasing transmission but a method of increasing the quality of care for HIV-positive individuals over time. Thus, it will be necessary to work with providers on continuing to give attention to the needs of ongoing patients.

HIV prevention needs to be multi-faceted and patients may feel that receiving information and support from various sources is beneficial. In this study, counsellors were most likely to offer patients a PHDP message, followed by clinical providers and lastly by pharmacy staff. Counsellors had almost two times higher odds of giving patients a PHDP message compared to clinical providers. These findings are not surprising given that in many health centres, there is a shortage of trained clinicians both in Mozambique as well as in other African contexts [57–59]. In such situations, counsellors tend to spend more time with patients addressing behavioural issues than clinicians. Additionally, in the Mozambican context, counsellors play an important role providing psycho-social support services. In various settings, counsellors have been found to be effective at delivering HIV prevention messages [60, 61]. However, research in the US has suggested that messages given by clinicians have the most impact on changing the behaviours of HIV clients [32]. The lower number of messages given by clinicians, although perhaps not surprising, is disappointing, given that messages from clinical providers have been shown to be effective at reducing high risk behaviours [62] and may carry more weight. Clinicians should continue to be encouraged to help their patients reduce risk behaviours and may be best placed to offer bio-medical messages about treatment for STIs and PMTCT, for example. However, due to the patient volume and complex needs of PLHIV, various cadres could offer HIV prevention messages so that comprehensive prevention that better meets the needs of patients is offered.

For pharmacies and pharmacy staff who are serving and supporting patients with HIV, optimal adherence is critical for improved health as well as prevention [63]. Our findings indicate that patients were unlikely to receive information about the importance of, or support for, adherence to ART during their pharmacy visits. Adherence, which is one of the PHDP messages, is a cornerstone of HIV prevention and care, and a visit to the pharmacy represents an opportunity for both adherence assessment and counselling. However, pharmacy and medication dispensing has not historically been utilized to its fullest benefit especially in low and middle income countries [64]. However, recent study findings indicate that for patients using pharmacies with HIV specific services or having HIV pharmacy staff involved in HIV care is associated both with improvements in ART adherence as well as facilitating an increase in the proportion of patients with suppressed viral load [65] and higher medication refill rates [63]. Also, in a recent review [65], in all but one study, the contribution of an HIV pharmacist to patient care was associated with clinically and statistically significant improvements in ART adherence. Supporting patients with HIV to achieve ART adherence is important for patients and because of the low documented number of PHDP messages given during pharmacy visits, this is an area that could be targeted for improvement in the delivery of PHDP. This represents a missed opportunity for pharmacy staff to offer an appropriate PHDP message, prevention education and behavioural support.

There are several limitations to our study. The pre-post design is vulnerable to a time bias (perhaps PHDP message delivery was improving due to an undocumented outside effect, unrelated to our training and materials). In this study, documentation of PHDP messages on the *Ficha de Avaliação Psicossocial e de Prevenção Positiva* was used as proxy for PHDP messages delivered. Clinicians may be delivering messages but may not document this delivery, despite training and recommendations (resulting in under-reporting). Alternatively, clinicians may be documenting message delivery which never occurred or which patients do not understand (resulting in over-reporting). If a clinician knows the patient, and believes a message will be of no value (for example, they know the person does not drink alcohol or is not sexually active) it is reasonably certain messages will not be provided. This will result in apparent under-delivery of messages, but would not result in a negative impact on the patient’s well-being.

## Conclusions

For the thousands of individuals living with or at risk for HIV in Mozambique, HIV prevention is paramount. Targeting HIV prevention efforts with individuals who are aware of their infection is one of the most effective HIV prevention approaches we have towards eliminating HIV transmission. An effective approach to preventing the transmission of HIV includes supporting prevention efforts at the point of clinical care. Our intervention improved PHDP message delivery in rural Mozambique, but despite intensive training using established materials and monthly follow-up with all providers, PHDP message delivery remained unacceptably low. We need to develop novel strategies to motivate health care providers to deliver these messages more consistently to all patients.
